# Non-Targeted Metabolomics Analysis of the Effects of Tyrosine Kinase Inhibitors Sunitinib and Erlotinib on Heart, Muscle, Liver and Serum Metabolism In Vivo

**DOI:** 10.3390/metabo7030031

**Published:** 2017-06-22

**Authors:** Brian C. Jensen, Traci L. Parry, Wei Huang, Amro Ilaiwy, James R. Bain, Michael J. Muehlbauer, Sara K. O’Neal, Cam Patterson, Gary L. Johnson, Monte S. Willis

**Affiliations:** 1 Department of Medicine, Division of Cardiology, University of North Carolina, Chapel Hill, NC 27599, USA; 2 McAllister Heart Institute, University of North Carolina, Chapel Hill, NC 27599, USA; traci_parry@med.unc.edu (T.L.P.); weih@email.unc.edu (W.H.); 3 Department of Pathology & Laboratory Medicine, University of North Carolina, Chapel Hill, NC 27599, USA; 4 Sarah W. Stedman Nutrition and Metabolism Center, Duke Molecular Physiology Institute, Duke University Medical Center, Durham, NC 27701, USA; amroilaiwy@gmail.com (A.I.); james.bain@duke.edu (J.R.B.); michael.muehlbauer@duke.edu (M.J.M.); sara.o’neal@duke.edu (S.K.O.); 5 Department of Medicine, Division of Endocrinology, Metabolism, and Nutrition, Duke University Medical Center, Durham, NC 27701, USA; 6 Presbyterian Hospital/Weill-Cornell Medical Center, New York, NY 10065, USA; cpatters@nyp.org; 7 Department of Pharmacology, University of North Carolina, Chapel Hill, NC 27599, USA; gary_johnson@med.unc.edu

**Keywords:** erlotinib, sorafenib, kinase inhibitors, cardiotoxicity, metabolomics, serum, liver, muscle, heart

## Abstract

*Background:* More than 90 tyrosine kinases have been implicated in the pathogenesis of malignant transformation and tumor angiogenesis. Tyrosine kinase inhibitors (TKIs) have emerged as effective therapies in treating cancer by exploiting this kinase dependency. The TKI erlotinib targets the epidermal growth factor receptor (EGFR), whereas sunitinib targets primarily vascular endothelial growth factor receptor (VEGFR) and platelet-derived growth factor receptor (PDGFR).TKIs that impact the function of non-malignant cells and have on- and off-target toxicities, including cardiotoxicities. Cardiotoxicity is very rare in patients treated with erlotinib, but considerably more common after sunitinib treatment. We hypothesized that the deleterious effects of TKIs on the heart were related to their impact on cardiac metabolism. *Methods:* Female FVB/N mice (10/group) were treated with therapeutic doses of sunitinib (40 mg/kg), erlotinib (50 mg/kg), or vehicle daily for two weeks. Echocardiographic assessment of the heart in vivo was performed at baseline and on Day 14. Heart, skeletal muscle, liver and serum were flash frozen and prepped for non-targeted GC-MS metabolomics analysis. *Results:* Compared to vehicle-treated controls, sunitinib-treated mice had significant decreases in systolic function, whereas erlotinib-treated mice did not. Non-targeted metabolomics analysis of heart identified significant decreases in docosahexaenoic acid (DHA), arachidonic acid (AA)/ eicosapentaenoic acid (EPA), O-phosphocolamine, and 6-hydroxynicotinic acid after sunitinib treatment. DHA was significantly decreased in skeletal muscle (quadriceps femoris), while elevated cholesterol was identified in liver and elevated ethanolamine identified in serum. In contrast, erlotinib affected only one metabolite (spermidine significantly increased). *Conclusions:* Mice treated with sunitinib exhibited systolic dysfunction within two weeks, with significantly lower heart and skeletal muscle levels of long chain omega-3 fatty acids docosahexaenoic acid (DHA), arachidonic acid (AA)/eicosapentaenoic acid (EPA) and increased serum O-phosphocholine phospholipid. This is the first link between sunitinib-induced cardiotoxicity and depletion of the polyunsaturated fatty acids (PUFAs) and inflammatory mediators DHA and AA/EPA in the heart. These compounds have important roles in maintaining mitochondrial function, and their loss may contribute to cardiac dysfunction.

## 1. Introduction

More than 90 tyrosine kinases have been implicated in the pathogenesis of malignant transformation and tumor angiogenesis [1,2]. Tyrosine kinase inhibitors (TKIs) have proven effective in cancer treatments by exploiting this kinase dependency [3]. The tyrosine kinase inhibitor sunitinib targets primarily vascular endothelial growth factor receptor (VEGFR) and platelet-derived growth factor receptor (PDGFR), while erlotinib targets the epidermal growth factor receptor (EGFR) [4]. Despite their specific targeting of tyrosine kinases, TKIs also impact the function of non-malignant cells with on- and off-target toxicities [4].

Recent population-based observational cohort studies have assessed the rates of cardiovascular disease after treatment with sunitinib and erlotinib [5]. Examination of 141,601 individual case safety reports extracted from from VigiBase^®^ (the World Health Organization database for individual case safety reports) for TKIs (including sunitinib and erlotinib) identified 2594 cardiac failure cases, with sunitinib among five TKIs (along with dasatinib, imatinib, bosutinib, and nilotinib) having a disproportionally increased risk of cardiac failure (1.67 95% CI 1.51, 1.84) [6]. Subsequent studies tested sunitinib and erlotinib on force-generating engineered heart tissues from neonatal rat heart cells [7]. After 96 h of incubation, a concentration and time-dependent decline in contractile force was identified with sunitinib, but not erlotinib, treatment [7]. This decline was associated with an impairment of autophagy and the appearance of autophagolysosomes. Studies directly testing cardiotoxicity of KIs in animals have found sunitinib cardiotoxic, but not erlotinib [8,9]. In contrast to sunitinib, most studies of erlotinib have not found it to be cardiotoxic. However, one study identified 11 erlotinib patients having ischemic heart disease [5]. In this population-based observational study with a 380-day median follow-up, 18 cases of ischemic heart disease (IHD) were identified, 11 which occurred in erlotonib patients (of 1046 total) and 5 in sunitinib-treated patients (9 of 430 total) [5]. These cases occurred predominantly in the late follow-up period and do not provide a direct causal link between erlotinib and IHD [5]. A recent meta-analysis of clinical trials using sunitinib (1077 people) identified an increased risk of heart failure (Relative Risk (RR) ratio of 4.3 with a number needed to harm (NNH) of 11) [10]. Neither sunitinib nor erlotinib was associated with an increased incidence of hypertension in these two studies [10].

Recent studies in human cardiomyocytes have identified cytotoxicity due to sunitinib, but not erlotinib, in vitro [11]. In these studies, sunitinib decreased cardiomyocyte viability, inhibited AMPK, increased lipid accumulation, disrupted beat pattern, and blocked hERG activity); in contrast, erlotinib demonstrated only minor changes (increased acetyl-CoA carboxylase (ACC) phosphorylation, the rate-limiting step in fatty acid biosynthesis), did not impact ROS, caspase, or lipid levels, and did not affect beat patterns [11]. Similarly, sunitinib treatment in vivo enhances myocardial expression of pro-inflammatory cytokines and enhances the expression of pro-fibrotic factors, while decreasing factors that degrade collagen [12]. Interestingly, treatment with L-carnitine, which is known to shuttle free fatty acids from the cytosol into mitochondria for beta oxidation and energy production, is protective against these effects of sunitinib [12]. Collectively, these human and animal data suggest that sunitinib is cardiotoxic, whereas erlotinib is cardiosafe, though the mechanisms underlying this difference are unclear.

In the present study, mice were treated with sunitinib or erlotinib to determine their effects on metabolism at the level of the heart, liver, skeletal muscle, and serum using a non-targeted metabolomics approach. We sought to determine whether metabolic signatures distinguish cardiotoxic sunitinib from cardiosafe erlotinib. 

## 2. Results

Sunitinib is an orally-delivered small molecule given in cycles of 50 mg per day for 4 weeks to maintain the therapeutic serum concentration (50–100 ng/mL) [13]. Dosing mice with 40 mg/kg yields comparable sunitinib concentrations and selectively inhibits VEGFR2 and PDGF receptor phosphorylation [14,15]. Erlotinib is also orally delivered and given daily (150 mg) 1–2 h after meals, resulting in C_max_ of ~1500 ng/mL, with a half-life of ~16 h [16]. In this study, we chose to treat mice with 50 mg/kg erlotinib daily, as this dosing achieves plasma concentrations within the therapeutic range for humans [17]. 

**Figure 1:**
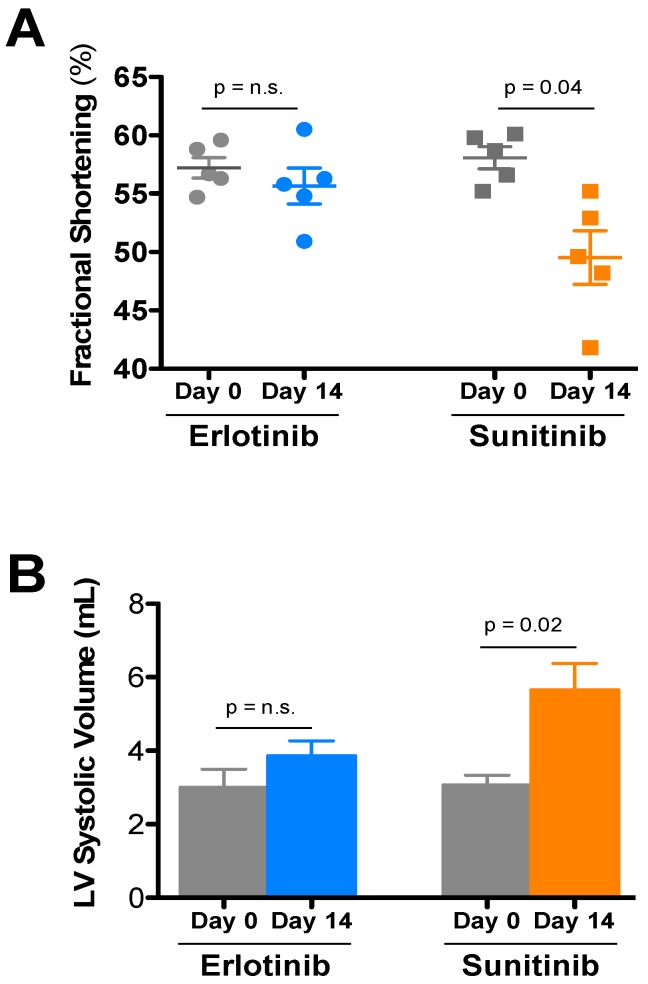
Echocardiographic analysis of the effects of the tyrosine kinase inhibitors erlotinib and sunitinib on cardiac function. FVB/N mice were treated with sunitinib (40 mg/kg), erlotinib (50 mg/kg), or vehicle daily for 2 weeks and serially echoed at baseline and after 2 weeks. (**A**) Fractional shortening % and (**B**) LV Volume (in Systole) at baseline and 14 days of erlotinib (blue), sunitinib (orange), or vehicle control (gray) treatment in vivo. A Student’s *t*-test was used to determine significance between groups (defined as *p* < 0.05). Values are expressed as mean values ± SE (*N* = 10/group).

After two weeks of treatment, wild-type female FVB/N mice treated mice treated with 40 mg/kg sunitinib were found to have impaired systolic function as compared to vehicle-treated mice, whereas 50 mg/kg erlotinib did not affect contractile function (Figure 1A). Beyond significant increases in LV diameter after sunitinib (Figure 1B), conscious echocardiography analysis revealed no other alterations (Table 1). This cardiotoxicity is consistent with previous studies demonstrating the much lower toxic threshold of sunitinib compared to erlotinib [7].

**Figure 2:**
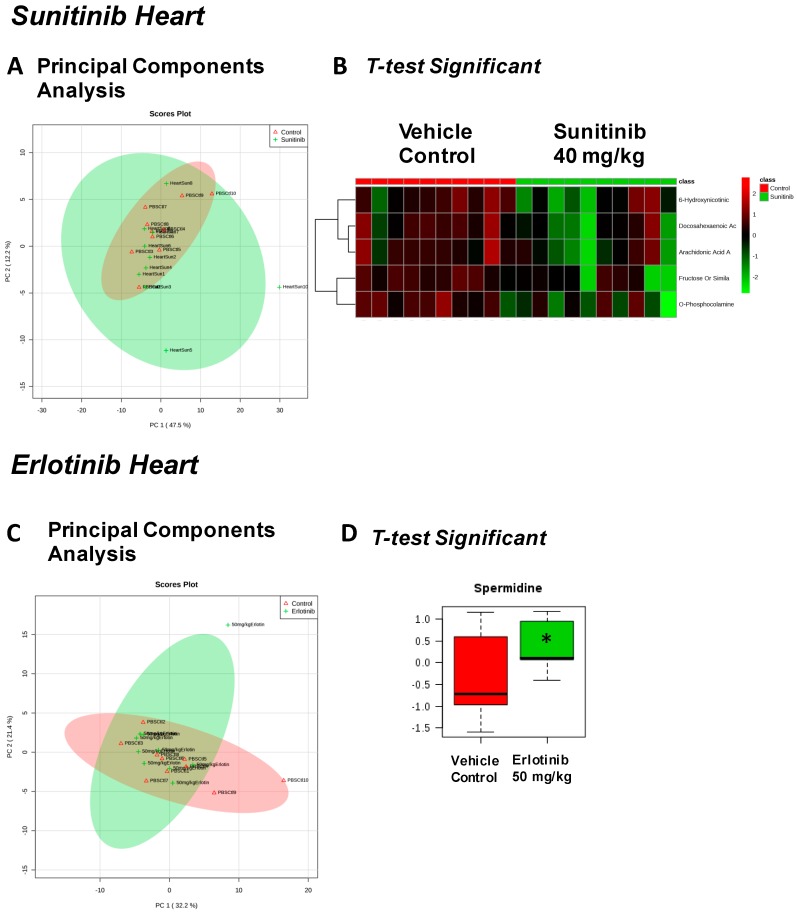
Significant metabolites identified in the heart 2 weeks after tyrosine kinase inhibitor (or vehicle control) treatment. PCA (principal components analysis) of metabolites identified in sunitinib-treated heart (**A**). *t*-test significant metabolites identified in sunitinib-treated heart (**B**). PCA (principal components analysis) of metabolites identified in erlotinib-treated heart (**C**). *t*-test significant metabolites identified in erlotinib-treated heart (**D**). *N* = 10/group.

We next assayed heart, liver, skeletal muscle (quadriceps femoris), and serum collected after 2 weeks of TKI treatment using non-targeted metabolomics analysis to explore whether metabolic alterations may have contributed to the observed effects on cardiac function. In the hearts of mice treated with sunitinib, 92 metabolites were identified (Figure S1, Table S1), revealing primarily overlap between the sunitinib and vehicle control-treated mice (Figure 2A), consistent with only 5 metabolites identified as significant by *t*-test (Figure 2B). In erlotinib-treated mice, 87 metabolites were identified (Figure S2, Table S2), with little resolution between the erlotinib and vehicle-treated mice by principal components analysis (PCA) (Figure 2C), and only one metabolite (spermidine) significantly increased by *t*-test (Figure 2D).

**Figure 3:**
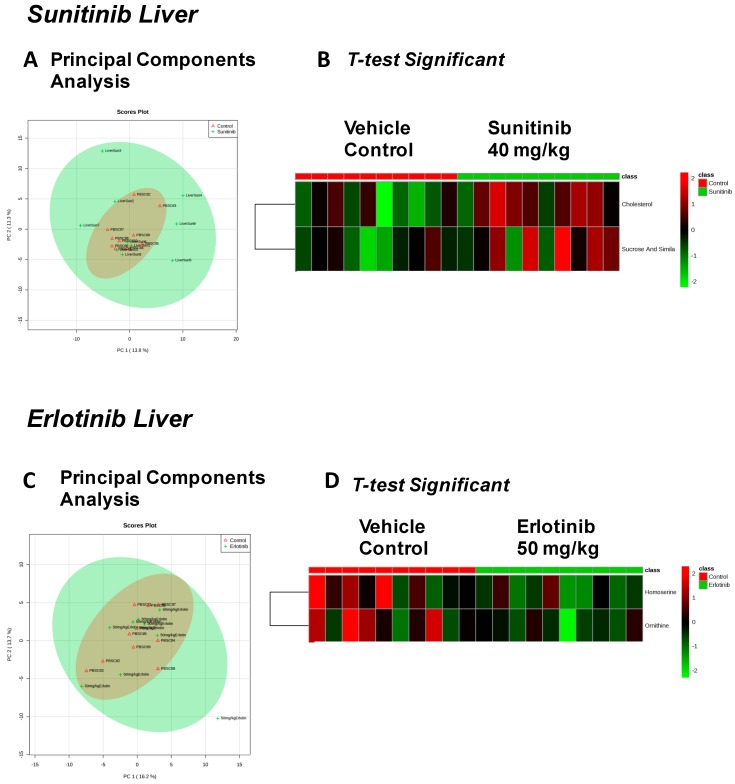
Significant metabolites identified in the liver 2 weeks after tyrosine kinase inhibitor (or vehicle control) treatment. PCA (principal components analysis) of metabolites identified in sunitinib-treated liver (**A**). *t*-test significant metabolites identified in sunitinib-treated liver (**B**). PCA (principal components analysis) of metabolites identified in erlotinib-treated liver (**C**). *t*-test significant metabolites identified in erlotinib-treated liver (**D**). *N* = 10/group.

Given reports of both sunitinib-related hepatic failure [18] and erlotinib-related hepatotoxicity [19,20], we investigated the metabolic effects of sunitinib and erlotinib on liver. We identified 115 metabolites in sunitinib-treated livers (Figure S3, Table S3) and 100 metabolites in erlotinib-treated livers (Figure S4, Table S4). With considerable overlap in the metabolic features of sunitinib-treated and vehicle-control treated livers (Figure 3A), only cholesterol and sucrose (and similar disaccharides) were elevated with sunitinib treatment (Figure 3B). PCA revealed considerable overlap between the liver metabolomes of erlotinib- and vehicle-treated mice (Figure 3C), with homoserine and ornithine significantly decreased with erlotinib treatment (Figure 3D). 

**Figure 4:**
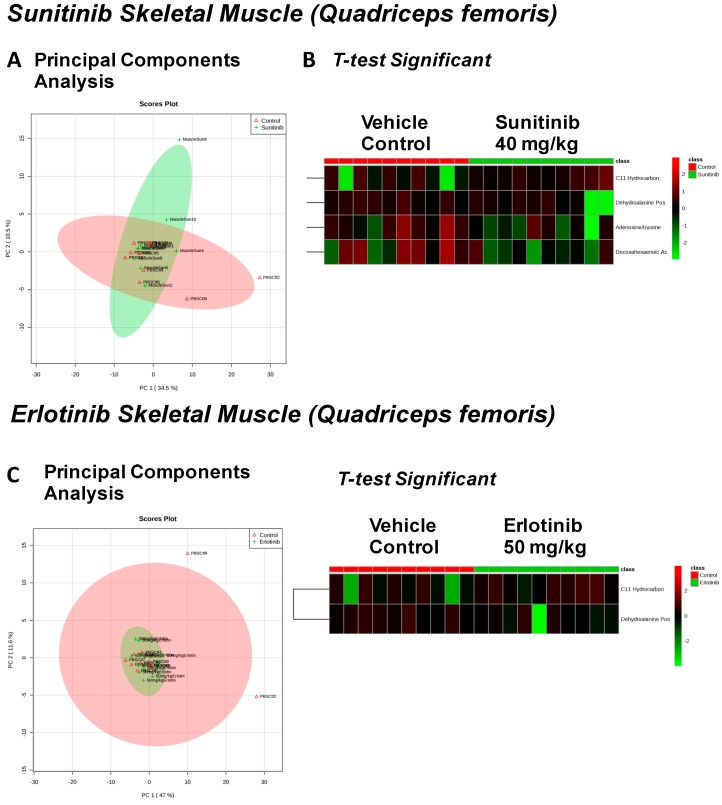
Significant metabolites identified in skeletal muscle 2 weeks after tyrosine kinase inhibitor (or vehicle control) treatment. PCA (principal components analysis) of metabolites identified in quadriceps femoris after sunitinib treatment (**A**). *t*-test significant metabolites identified in quadriceps femoris after sunitinib treatment (**B**). PCA (principal components analysis) of metabolites identified in quadriceps femoris after erlotinib treatment (**C**). *t*-test significant metabolites identified in quadriceps femoris after erlotinib treatment (**D**). *N* = 10/group.

The effects of sunitinib treatment on skeletal muscle (quadriceps femoris) were investigated, where we identified 92 metabolites (Figure S5, Table S5) distinguished into two overlapping groups by PCA analysis (Figure 4A), and four significantly altered metabolites identified (Figure 4B), including significant decreases in dehydroalanine, adenosine, and docosahexaenoic acid. Eighty-three metabolites were identified from ertlotinib-treated quadriceps femoris (Figure S6, Table S6), again largely overlapping with vehicle treatment (Figure 4C), with two significantly altered metabolites identified by *t*-test, dehydroalanine (likely a GC/MS artifact of cysteine degradation) and a C11 hydrocarbon (Figure 4D).

**Figure 5:**
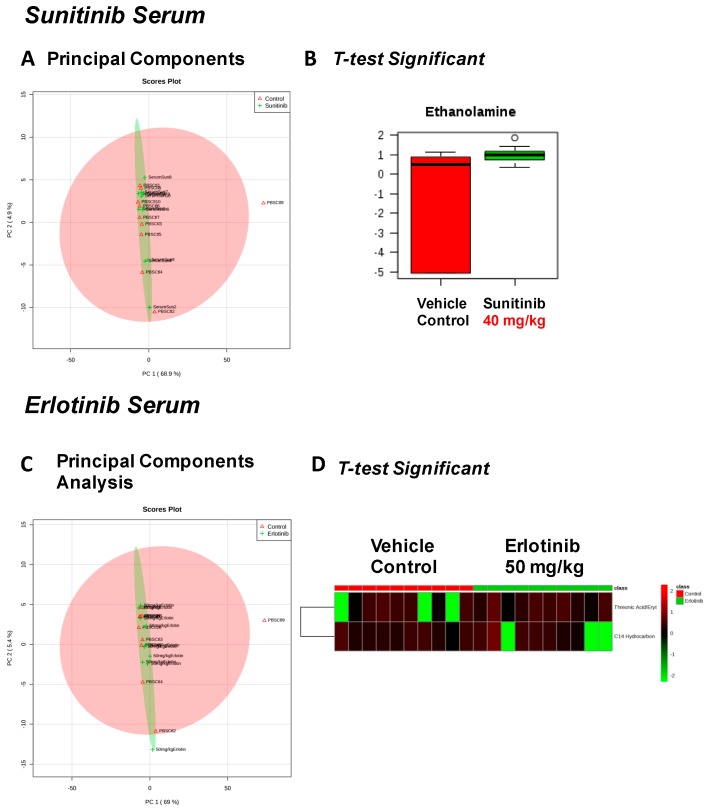
Significant serum metabolites identified after 2 weeks of tyrosine kinase inhibitor (or vehicle control) treatment. PCA (principal components analysis) of serum metabolites from sunitinib-treated mice (**A**). *t*-test significant metabolites identified in serum from sunitinib-treated mice (**B**). PCA (principal components analysis) of serum metabolites from erlotinib-treated mice (**C**). *t*-test significant metabolites identified in serum from erlotinib-treated mice (**D**). *N* = 10/group.

In serum from sunitinib- and erlotinib-treated mice, we identified 125 metabolites (Figure S7/Table S7, Figure S8/Table S8, respectively). Sunitinib-treated serum had few changes from vehicle control-treated mice (Figure 5A), with ethanolamine being the only significantly increased metabolite (Figure 5B). Similarly, the metabolites identified in the erlotinib-treated serum largely overlapped those of vehicle controls (Figure 5C), with only two significantly altered metabolites, including increased threonic acid and C14 hydrocarbon (Figure 5D).

## 3. Discussion

Sunitinib inhibits multiple tyrosine receptor kinases including PDGFR, VEGFR, and CD117 (c-KIT) to reduce tumor burden through decreased vascularization and enhanced cancer cell apoptosis. Sunitinib has been approved by the FDA for the treatment of renal cell carcinoma and imatinib-resistant gastrointestinal stromal tumor (GIST). Sunitinib cardiotoxicity has been reported in multiple clinical trials, but the specific mechanisms are not clearly understood. Erlotinib is a tyrosine kinase inhibitor that targets the EGFR in locally advanced or metastatic non-small cell lung cancer (NSCLC) that has failed a prior chemotherapy regimen. It has also been FDA approved for combination use with gemcitabine for the treatment of locally advanced, unresectable, or metastatic pancreatic cancer. Erlotinib is not clearly associated with cardiotoxicity.

Recent studies of the effects of sunitinib on isolated rodent hearts revealed increases in TNF-α and TnT in the perfusion buffer at the same time as impaired cardiac function, indicating direct cardiotoxicity [8]. In contrast, treating isolated hearts with erlotinib did not elicit increases in any of the biomarkers investigated (BNP, IL6, TNF-α, cTnT, cTnI) [8]. Other studies have also reported that sunitinib affects cardiac function, linked to an effect on autophagic flux, while erlotinib does not [7]. In the present study, we also find evidence that sunitinib is cardiotoxic, whereas erlotinib is cardiosafe in mice treated with doses comparable to those used in humans (Figure 1). We identified significant changes in 9 heart and skeletal muscle metabolites when treated with sunitinib (Table 2), compared to 3 significantly altered metabolites in erlotinib-treated heart and skeletal muscle (Table 3). As all mice were the same sex, age, and strain, and all samples were treated and analyzed identically, we attribute these differences to the distinct metabolic effects of the two TKIs.

Mice treated with sunitinib had significantly lower heart and skeletal muscle levels of the long chain omega-3 fatty acids docosahexaenoic acid (DHA) and arachidonic acid (AA) / eicosapentaenoic acid (EPA) (summarized in Table 2). Recent studies have confirmed the importance of omega-3 PUFAs in reducing cardiovascular disease and associated inflammation [21,22]. Significant decreases in cardiac O-phosphocoline were identified with sunitinib treatment (Table 2). O-Phosphocholine is the head group of the major concentrated phospholipid in human plasma (76% phosphocholine, 17% phosphoethanolamine) [23] and is the most abundant phospholipid in human erythrocytes [24]. As such, phosphocholine and phosphoethanolamine are the biggest reservoirs of dietary n-6 and n-3 PUFAS involved in inflammatory response. DHA and AA are critical regulators of cardiomyocyte membranes, responsible for the maintenance of cholesterol homeostasis [25]. The long-chain omega-3 fatty acid DHA (22:6n-3) is best known for its cardioprotective properties, due in part to its incorporation into cell membranes, resulting in a direct effect on calcium channels and their role in eicosanoid metabolism [26,27,28]. The mechanism by which AA / EPA and DHA are immunomodulatory seems to be due to derivatives of these PUFAs [29], including bioactive lipid mediators called resolvins, protectins, and marescins, which have potent anti-inflammatory and immunoregulatory action in vitro and in vivo [30,31]. While the sunitinib-induced decreases in these potent anti-inflammatory mediators cannot be directly linked to the observed cardiotoxicity, these findings do suggest a novel link between sunitinib-induced cardiotoxicity and alterations in inflammation through its effects on DHA, arachidonic acid, and O-phosphocolamine (aka phosphoethanolamine).

In the present study, there is a significant elevation in liver cholesterol levels after sunitinib treatment (Table 2). Previous studies have made the clinical observation that sunitinib treatment causes hyperlipidemia in patients with metastatic renal cell carcinoma [32]. While the new-onset hyperlipidemia was found to be higher than in matched controls [32], no clear mechanism has been described. The results in the present study identify for the first time that sunitinib increases liver cholesterol in vivo, suggesting that sunitinib’s direct effects on hepatic cholesterol biosynthesis may contribute to the hyperlipidemia recently reported in patients. 

Sunitinib-treated rodents demonstrated elevated ethanolamine in the serum (Table 2). In serum, ethanolamine is converted to phosphoethanolamine by ethanolamine kinase, which constitutes the head group of the major reservoir of phospholipids in human plasma [23]. The link between sunitinib treatment and serum ethanolamine is not clear, but may reflect a disrupted phospholipid metabolism in tissues (decreased AA/EPA, DHA, O-phosphocholine) resulting in elevated ethanolamine in the serum, and/or may reflect defects in the conversion of ethanolamine to phosphoethanolamine in the serum [33].

Our studies were conducted only in female mice, hence we cannot evaluate whether sex influences the response to TKIs. Nevertheless, our findings indicate distinct metabolic responses to sunitinib and erlotinib and suggest that metabolic derangements may play causative roles in the cardiotoxicity of sunitinib.

## 4. Materials and Methods 

### 4.1. Animals, Experimental Design, Drug Delivery, and Harvest 

Mice were 10-week old FVB/N females (UNC IACUC Approval Number 15-013). They were fed the usual animal care diet per the UNC Animal Care facility and were gavaged daily by UNC Lineberger Animal Models Core staff with erlotinib 50 mg/kg/day or sunitinib 40 mg/kg/day (LC Laboratories E-4007 and S-8803) solubilized in dimethyl sulfoxide (DMSO). DMSO concentration in vehicle control was the same as in the group with the highest TKI/DMSO concentration. At baseline and on Day 14, 5 mice from each group underwent conscious echocardiography as previously described [34,35]. Mice were sacrificed under deep isoflurane anesthesia, blood was collected by cardiac puncture and clotted, and 100–200 μL serum was snap-frozen in liquid nitrogen. The heart was dissected, the atria and great vessels were excised, and blood was cleared from the ventricles in cold PBS. Liver and quadriceps femoris muscle were also excised. Excess liquid was blotted from all tissues, and they were snap-frozen in liquid nitrogen. 

### 4.2 Non-Targeted Metabolomics Determination by GC–MS Instrumentation 

Left ventricular tissue was flash frozen in liquid nitrogen, weighed (25–50 mg wet weight), then placed in buffer (50% acetyl-nitrile, 50% water, 0.3 % formic acid) at a standard concentration of 25 mg/475 μL buffer and fully homogenized on ice for 20–25 s and placed on dry ice and stored at −80 °C. Samples were then analyzed by GC/MS as previously described [36]. The raw, transformed, and sorted data used is found in Supplemental Table S1. Three treatment groups (vehicle control, soratinib, erlotinib) each contained ten biological replicate samples (each with heart, liver, muscle, and serum analyzed for a total of 120 tissues from 30 total animals analyzed). 

### 4.3 Metabolomic Statistical Analyses 

Metaboanalyst (v3.0) run on the statistical package R (v2.14.0) used metabolite peak areas (as representative of concentration) [37,38,39]. These data were scaled using a Pareto scaling feature. To detect a systemic metabolic signature of the sorafenib treatment, a *t*-test was performed using Metaboanalyst v3.0. *t*-test significant metabolites (*p* < 0.05) were matched to metabolomics pathways using the Pathway Analysis and Enrichment Analysis features in Metaboanalyst 3.0. Only metabolites identified and detected in all groups were included in the statistical analysis. For the sunitinib-treated mice, metabolites were excluded if there were more than 3 individual biological replicates without a detectable metabolite (of 10 total heart, liver, muscle, or serum per group). For the erlotinib-treated mice, heart, liver, or muscle metabolites were excluded if there were more than 2 individual biological replicates without a detectable metabolite (of 10 total per group); serum metabolites were excluded if 3 or more values were not detected. The remaining missing values were imputed by replacing with ½ of the lowest minimum value via Metaboanalyst [37,38,39]. The raw data from this study is accessible through the NIH Common Fund’s Data Repository and Coordinating Center (supported by NIH grant, U01-DK097430) website, http://www.metabolomicsworkbench.org. All data from this study are available in Table S1. All data are shown as mean +/− SEM, unless otherwise indicated. 

### 4.4 Other Statistical Analyses. 

All data is shown as mean +/− SEM, unless otherwise indicated. Differences between two groups were compared with the Student’s *t*-test or the Mann-Whitney test for nonparametric data, while comparisons between >2 groups used a One-Way ANOVA. Post-hoc analysis was performed using a *t*-test with the Bonferroni adjustment method using Prism 7.0 (GraphPad Software, Inc., La Jolla, CA, USA).

## Figures and Tables

**Table 1:** Echocardiographic parameters after erlotinib or sunitinib treatment.

Treatment	Day	HR	LVIDd	LVIDs	FS	LVd vol	LVs vol	IVSd	PWd
**Sunitinib (5)**	Day 0	684 ± 14	2.76 ± 0.06	1.16 ± 0.04	58.1 ± 0.9	28.6 ± 1.5	3.08 ± 0.26	0.93 ± 0.01	0.95 ± 0.04
	Day 14	683 ± 13	2.88 ± 0.08	1.45 ± 0.07 *	49.5 ± 2.3	31.7 ± 2.1	5.66 ± 0.71 *	0.87 ± 0.02	0.95 ± 0.06
**Erlotinib (5)**	Day 0	672 ± 23	2.66 ± 0.11	1.14 ± 0.06	57.2 ± 0.9	26.3 ± 2.7	3.00 ± 0.49	0.85 ± 0.05	0.96 ± 0.01
	Day 14	668 ± 12	2.84 ± 0.08	1.26 ± 0.05	55.7 ± 1.5	30.7 ± 2.1	3.86 ± 0.40	0.95 ± 0.03	1.00 ± 0.07

Echocardiography was performed on un-anesthetized mice (*N* per group). All values are the mean ± SEM; * *p* < 0.05 vs. baseline. FS = fractional shortening (%); HR = heart rate (beats per minute); IVSd = interventricular septal thickness, diastole (cm); LVd vol = left ventricular diastolic volume (mL); LVs vol = left ventricular systolic volume (mL); LVIDd = left ventricular internal diameter, diastole (cm); LVIDs = left ventricular internal diameter, systole (cm); LVm = LV mass, calculated; PWd = posterior wall, diastole (cm).

**Table 2:** *t*-test significant metabolites in sunitinib-treated tissues compared to vehicle control treated. Color-matched metabolites are highlighted if they were found in two or more tissues (blue) or in the same tissue by both drugs (red).

Sunitinib Treatment			
Heart	Liver	Skeletal Muscle	Serum
Fructose Or Similar Ketohexose (↓)	Cholesterol (↑)	Adenosine/Inosine (↓)	Ethanolamine (↑)
Docosahexaenoic Acid (DHA) (↓)	Sucrose and Similar Disaccharides (↑)	Docosahexaenoic Acid (DHA) (↓)	
Arachidonic Acid Also Eicosapentaenoic Acid (EPA) (↓)		C11 Hydrocarbon (↓)	
O-Phosphocolamine (↓)		Dehydroalanine Possibly From Cysteine (↓)	
6-Hydroxynicotinic Acid (↓)			

**Table 3:** *t*-test significant metabolites in erlotinib-treated tissues compared to vehicle control treated. Color-matched metabolites are highlighted if they were found in two or more tissues (blue) or in the same tissue by both drugs (red).

Erlotinib Treatment			
Heart	Liver	Skeletal Muscle	Serum
Spermidine (↑)	Homoserine (↓)	C11 Hydrocarbon (↓)	Threonic Acid/Erythronic Acid (↑)
	Ornithine (↓)	Dehydroalanine Possibly From Cysteine (↓)	

## Supplementary Materials

The following are available online at www.mdpi.com/2218-1989/7/3/31/s1. Figure S1: Heat map of all cardiac metabolites identified by GC-MS in sunitinib-treated animals; Figure S2: Heat map of all cardiac metabolites identified by GC-MS in erlotinib-treated animals; Figure S3: Heat map of all liver metabolites identified by GC-MS in sunitinib-treated animals; Figure S4: Heat map of all liver metabolites identified by GC-MS in erlotinib-treated animals; Figure S5: Heat map of all quadriceps femoris metabolites identified by GC-MS in sunitinib-treated animals; Figure S6: Heat map of all quadriceps femoris metabolites identified by GC-MS in erlotinib-treated animals; Figure S7: Heat map of all serum metabolites identified by GC-MS in sunitinib-treated animals; Figure S8: Heat map of all serum metabolites identified by GC-MS in erlotinib-treated animals; Tables S1–S8: Non-targeted metabolomics performed on serum from mice treated with vehicle, sunitinib (40 mg/kg) or erlotinib (50 mg/kg).

## Abbreviations

DHA	docosahexaenoic acid
EPA	eicosapentaenoic acid
EGFR	epidermal growth factor receptor
IHD	ischemic heart disease
TKI	tyrosine kinase inhibitor
VEGFR	vascular endothelial growth factor receptor
