# Supplementary material for: Non-Targeted Metabolomics Analysis of the Effects of Tyrosine Kinase Inhibitors Sunitinib and Erlotinib on Heart, Muscle, Liver and Serum Metabolism In Vivo
Source: Metabolites. 2017 Jun 22;7(3):31. doi: 10.3390/metabo7030031 (PMC5618316; doi:10.3390/metabo7030031)
Supplement: Supplementary file 1 [file metabolites-07-00031-s001.zip › metabolites-07-00031-supplementary-Figure S1-8.pdf]

Supplementary Materials: Non-Targeted Metabolomics Analysis of the Effects of Tyrosine Kinase Inhibitors Sunitinib and Erlotinib on Heart, Muscle, Liver and Serum Metabolism *In Vivo*

Brian C. Jensen [1,*], Traci L, Parry, Wei Huang, Amro Ilaiwy, James R. Bain, Michael J. Muehlbauer, Sara K. O'Neal, Cam Patterson, Gary L. Johnson, Monte S. Willis

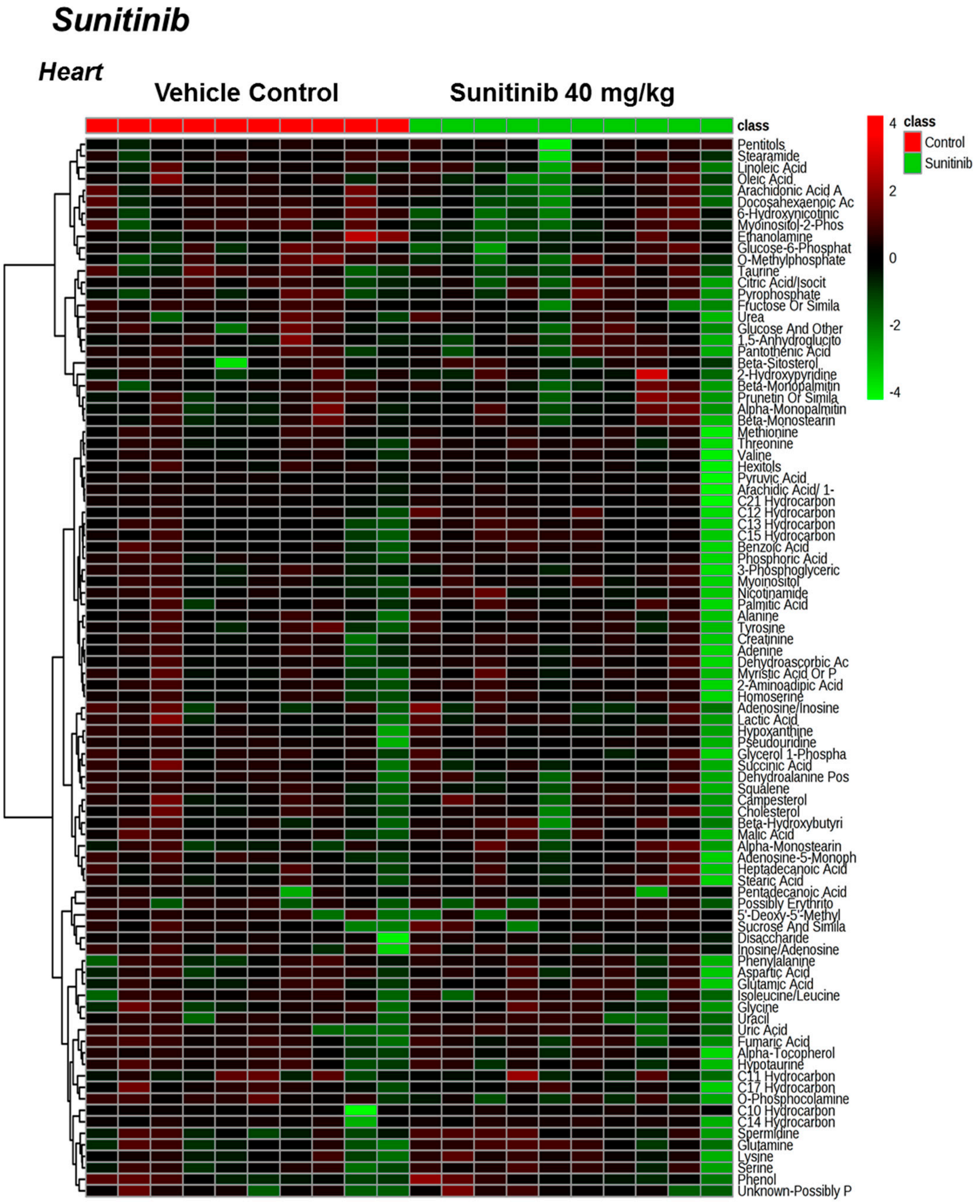

Figure S1. Heat map of all cardiac metabolites identified by GC-MS in sunitinib-treated animals.

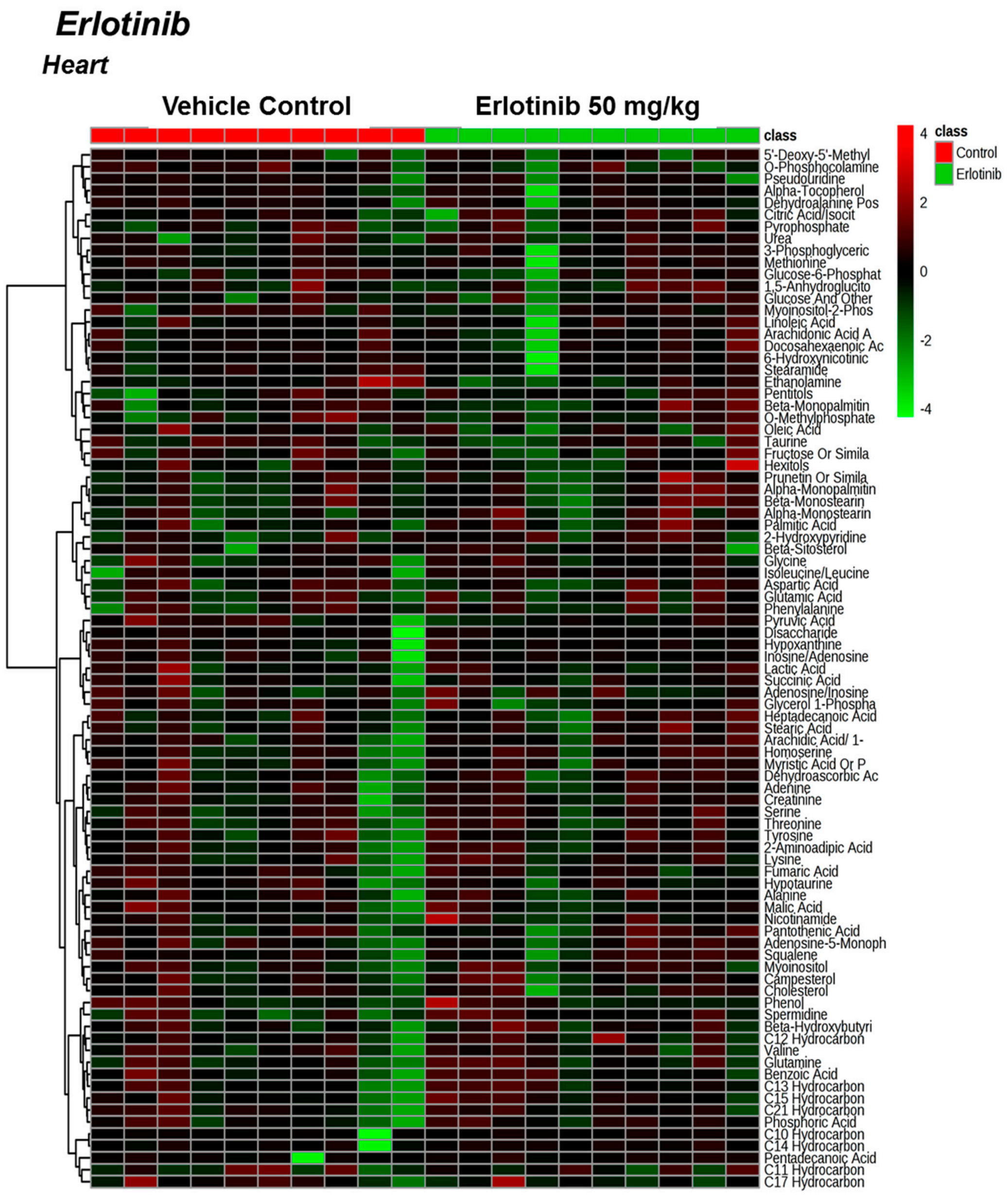

Figure S2. Heat map of all cardiac metabolites identified by GC-MS in erlotinib-treated animals.

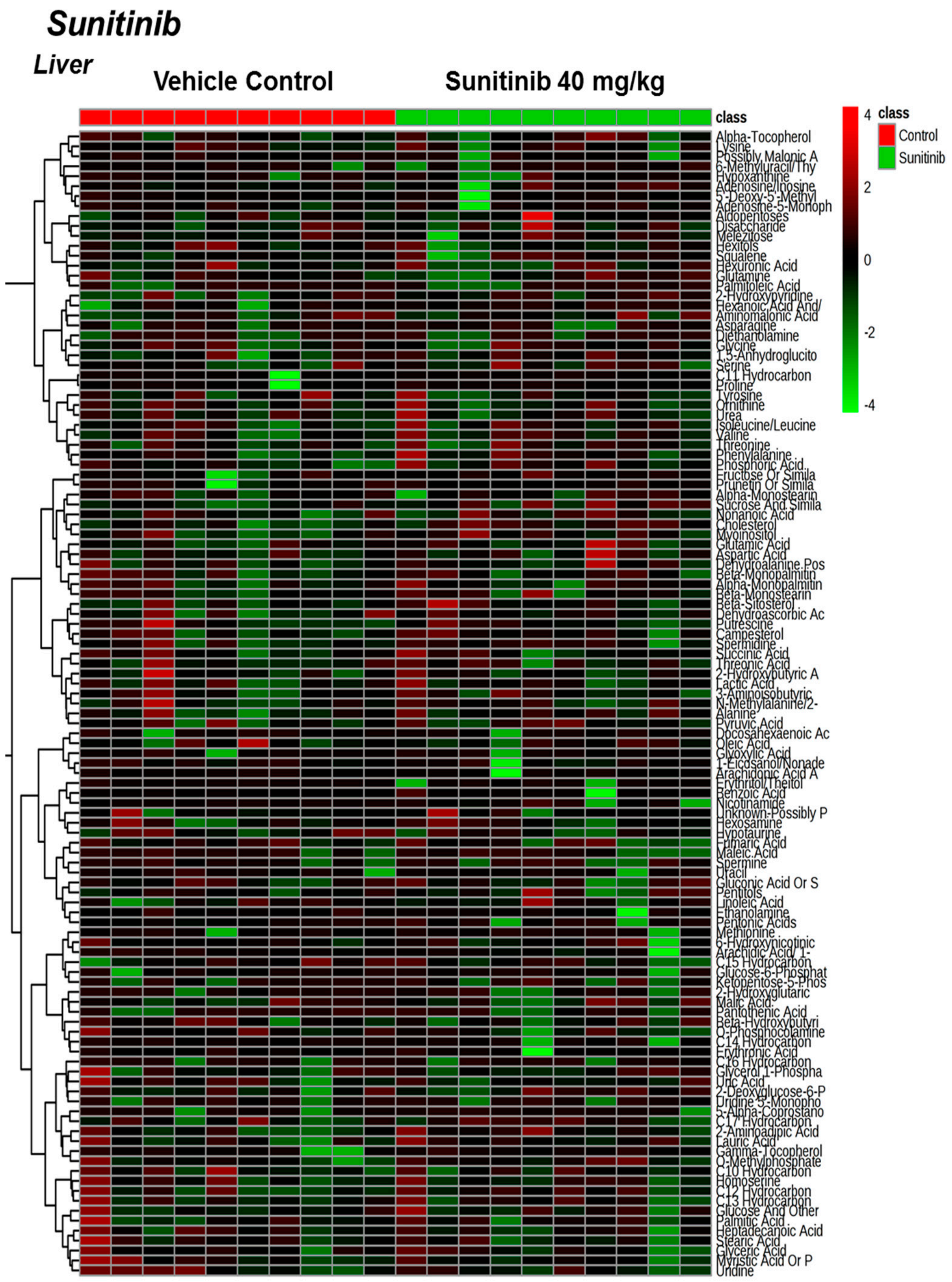

Figure S3. Heat map of all liver metabolites identified by GC-MS in sunitinib-treated animals.

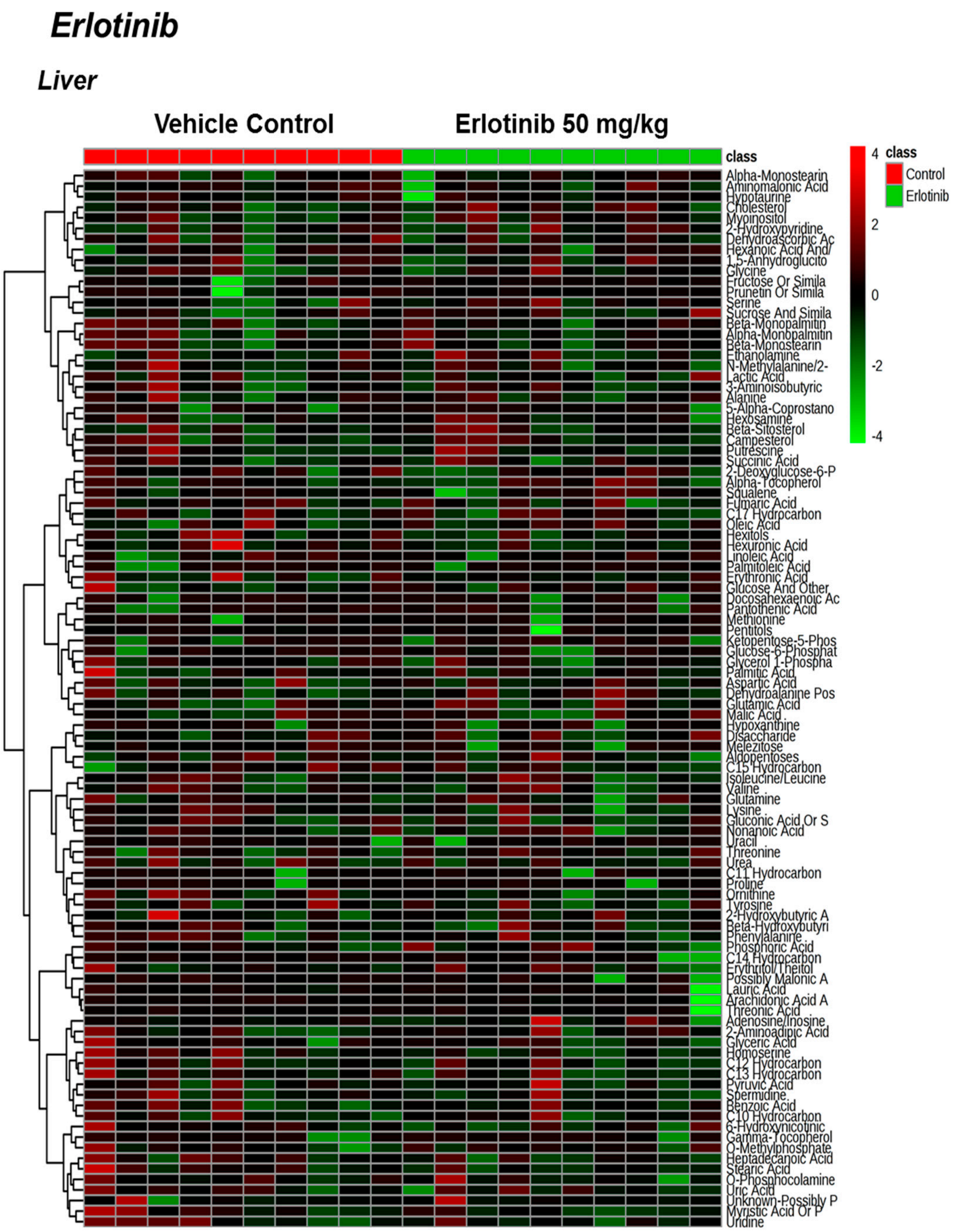

Figure S4. Heat map of all liver metabolites identified by GC-MS in erlotinib-treated animals.

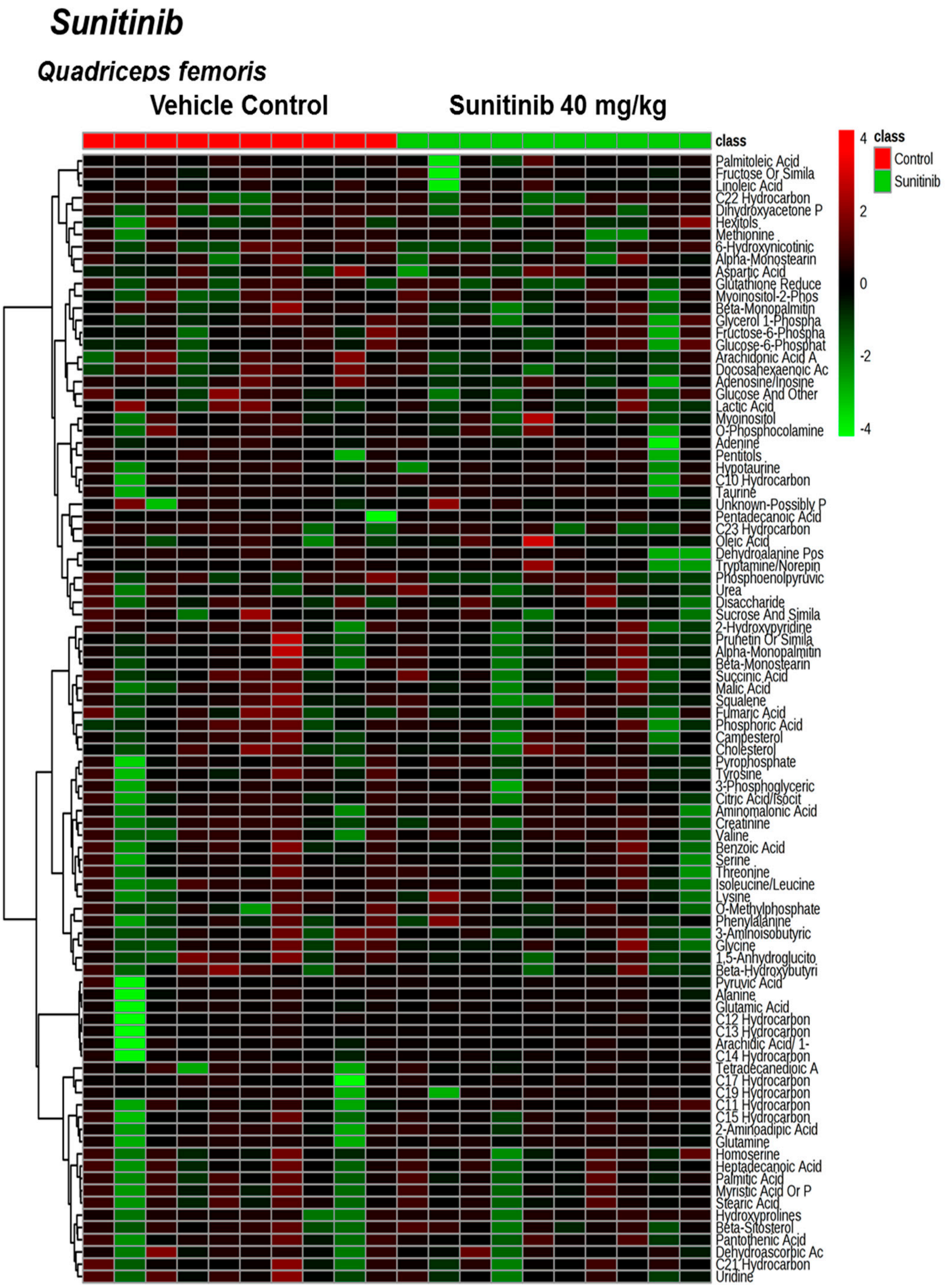

Figure S5. Heat map of all quadriceps femoris metabolites identified by GC-MS in sunitinib-treated animals.

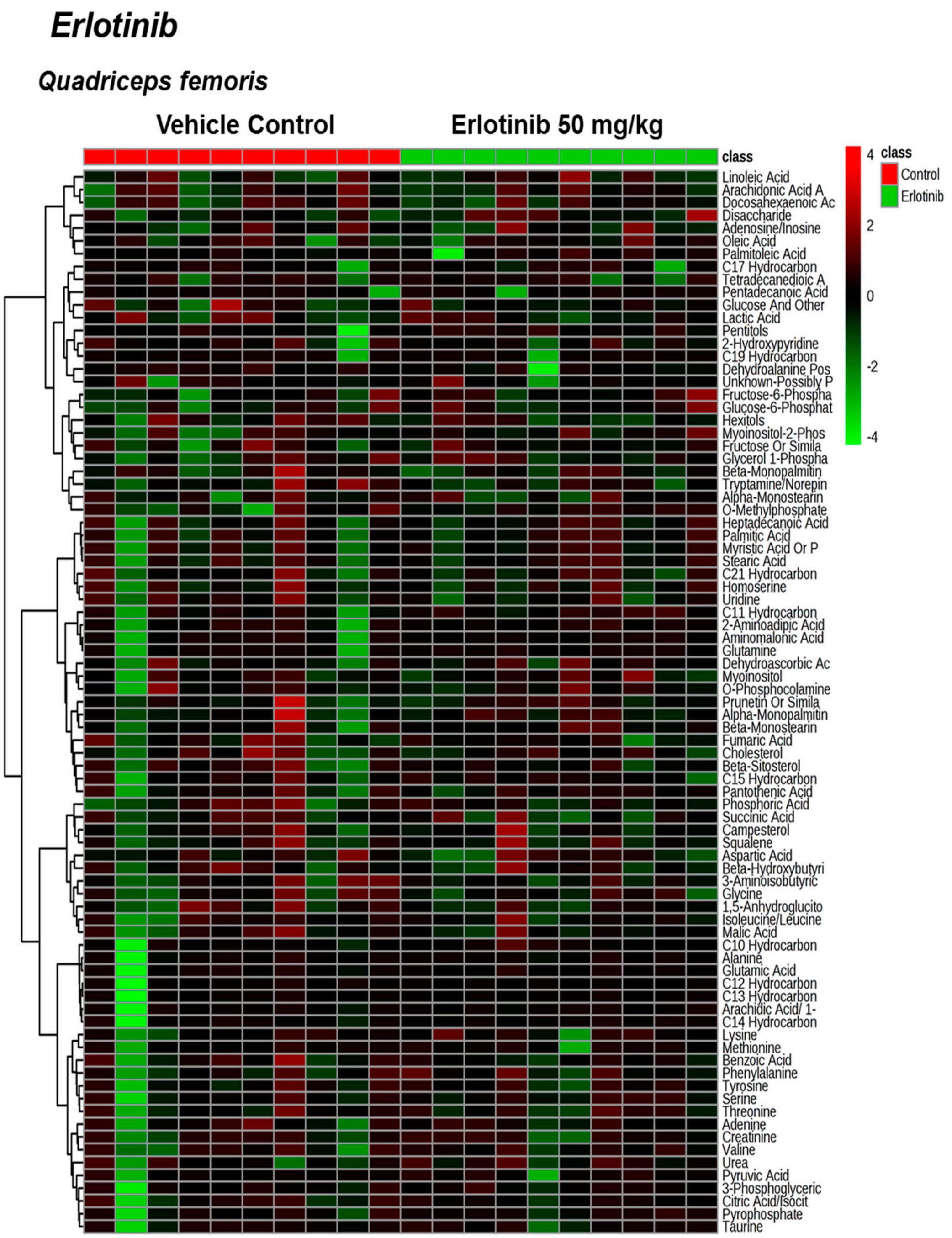

Figure S6. Heat map of all quadriceps femoris metabolites identified by GC-MS in erlotinib-treated animals.

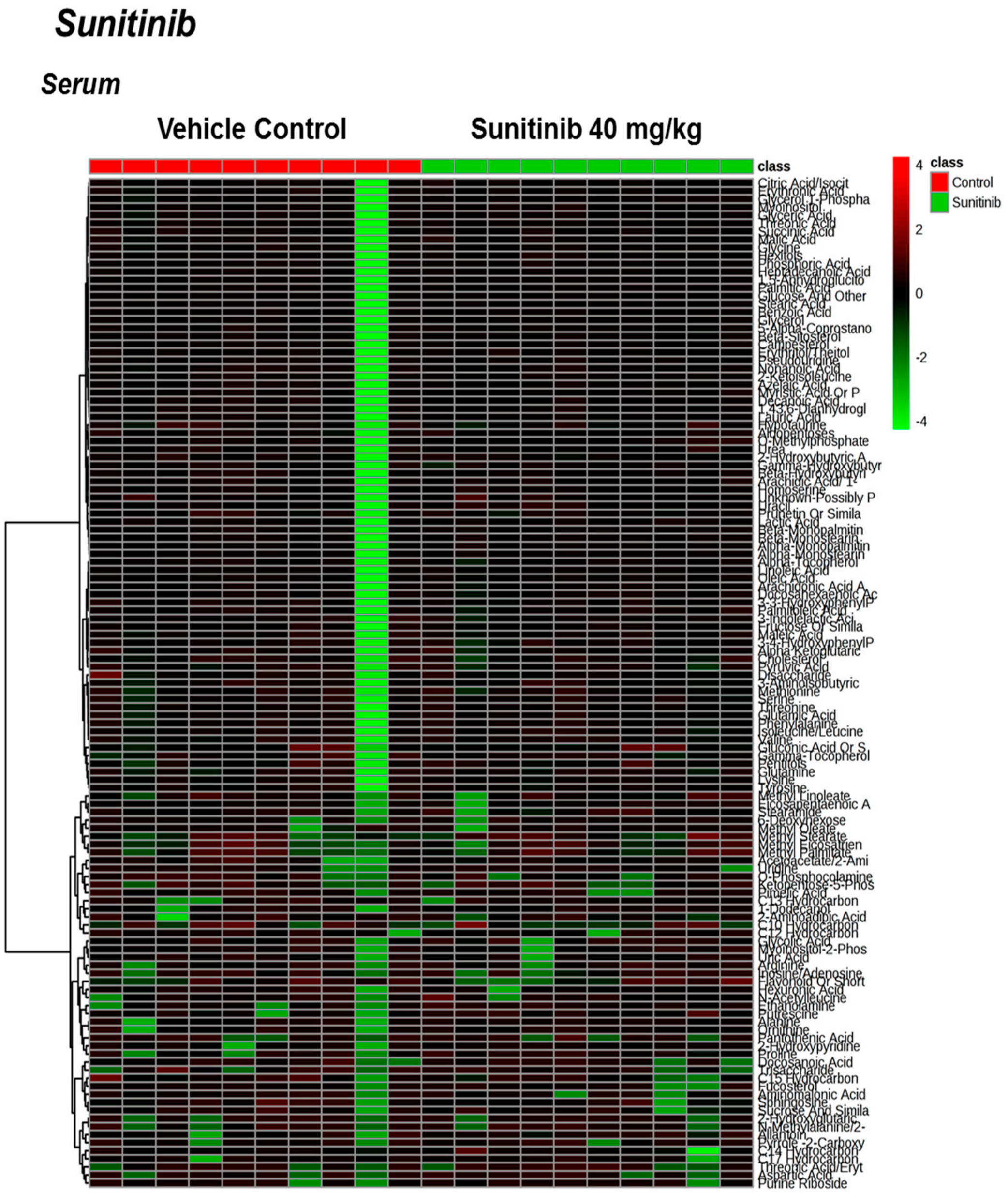

Figure S7. Heat map of all serum metabolites identified by GC-MS in sunitinib-treated animals.

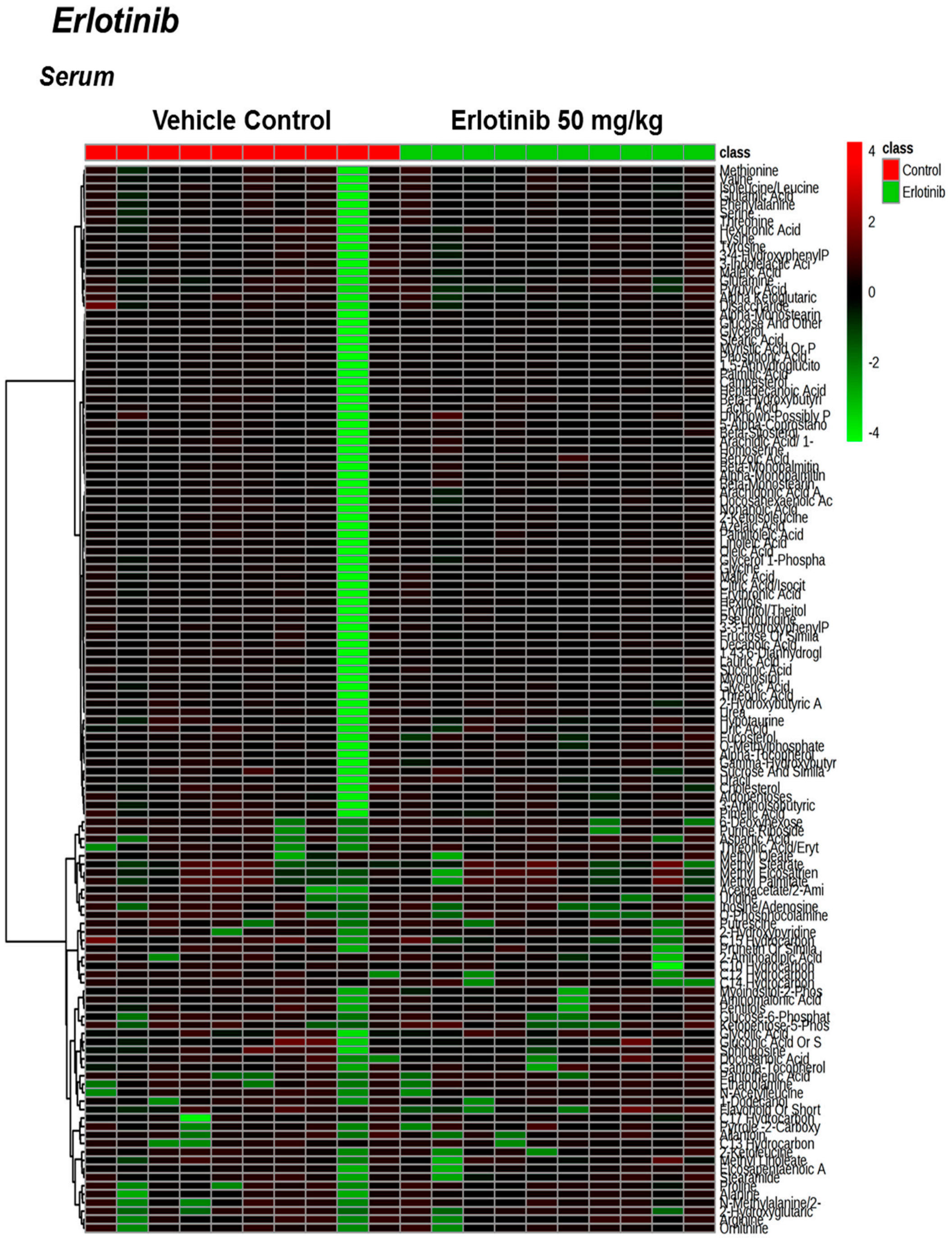

Figure S8. Heat map of all serum metabolites identified by GC-MS in erlotinib-treated animals.
